# E3 ligase HUWE1 promotes PDGF D-mediated osteoblastic differentiation of mesenchymal stem cells by effecting polyubiquitination of β-PDGFR

**DOI:** 10.1016/j.jbc.2022.101981

**Published:** 2022-04-25

**Authors:** Tri Pham, Abdo J. Najy, Hyeong-Reh C. Kim

**Affiliations:** Department of Pathology, Wayne State University School of Medicine and the Barbara Ann Karmanos Cancer Institute, Detroit, Michigan, USA

**Keywords:** human bone marrow mesenchymal stem cells, osteoblast differentiation, adipocyte differentiation, PDGF D, β-PDGFR, HUWE1, ubiquitination, ALP, alkaline phosphatase, BMP, bone morphogenetic protein, BMSC, bone marrow MSC, C/EBPα, CCAAT/enhancer binding protein alpha, CST, Cell Signaling Technologies, CM, conditioned media, DD, double-distilled, ERK, extracellular signal–regulated kinase, FABP4, fatty acid–binding protein 4, FDR, false discovery rate, GAP1, GTPase-activating protein 1, hBMSC, human bone marrow mesenchymal stem cell, HUWE1, HECT, UBA, and WWE domain–containing protein 1, IF, immunofluorescence, IP, immunoprecipitation, JNK, c-Jun N-terminal kinase, KD, knockdown, MSC, mesenchymal stem cell, PDGF, platelet-derived growth factor, PDGF D, platelet-derived growth factor D, β-PDGFR, PDGF receptor beta, PFA, paraformaldehyde, RhoA, Ras homolog family member A, RIPA, radioimmunoprecipitation assay, ROCK, Rho-associated protein kinase, rPDGF, recombinant PDGF, TGF-β, transforming growth factor-β, USP2, ubiquitin-specific peptidase 2

## Abstract

Mesenchymal stem cells (MSCs) are adult stem cell populations and exhibit great potential in regenerative medicine and oncology. Platelet-derived growth factors (PDGFs) are well known to regulate MSC biology through their chemotactic and mitogenic properties. However, their direct roles in the regulation of MSC lineage commitment are unclear. Here, we show that PDGF D promotes the differentiation of human bone marrow mesenchymal stem cells (hBMSCs) into osteoblasts and inhibits hBMSC differentiation into adipocytes. We demonstrate that PDGF D-induced β-actin expression and polymerization are essential for mediating this differential regulation of osteoblastogenesis and adipogenesis. Interestingly, we found that PDGF D induces massive upward molecular weight shifts of its cognate receptor, PDGF receptor beta (β-PDGFR) in hBMSCs, which was not observed in fibroblasts. Proteomic analysis indicated that the E3 ubiquitin ligase HECT, UBA, and WWE domain–containing protein 1 (HUWE1) associates with the PDGF D-activated β-PDGFR signaling complex in hBMSCs, resulting in β-PDGFR polyubiquitination. In contrast to the well-known role of ubiquitin in protein degradation, we provide evidence that HUWE1-mediated β-PDGFR polyubiquitination delays β-PDGFR internalization and degradation, thereby prolonging AKT signaling. Finally, we demonstrate that HUWE1-regulated β-PDGFR signaling is essential for osteoblastic differentiation of hBMSCs, while being dispensable for PDGF D-induced hBMSC migration and proliferation as well as PDGF D-mediated inhibition of hBMSC differentiation into adipocytes. Taken together, our findings provide novel insights into the molecular mechanism by which PDGF D regulates the commitment of hBMSCs into the osteoblastic lineage.

Mesenchymal stem cells (MSCs) are adult stem cell populations that exhibit strong immunomodulatory properties, regenerative capabilities, and differentiation potential into diverse cell types (1, 2). MSCs have been shown to home to sites of injured tissues where they facilitate tissue regeneration (3, 4). MSCs are also recruited by cancer cells and promote cancer progression and metastasis (5). These characteristics have generated significant interest in MSC biology in the context of regenerative medicine and oncology (1, 2). Physical, chemical, and biological factors have been extensively investigated to define microenvironmental stimuli that regulate MSC biology. Among those, transforming growth factor-β (TGF-β) and bone morphogenetic proteins (BMPs) are thought to play key roles in bone marrow MSC (BMSC) differentiation into bone cells (6, 7, 8). While BMPs and TGF-β were shown to induce stem cell differentiation and commitment to osteoprogenitor cells, preclinical and clinical studies showed marginal effects on bone regeneration when singly administered, and their clinical use is limited because of the requirement of supraphysiological concentrations for bone formation (9). Interestingly, receptor tyrosine kinase signaling was shown to enhance osteogenic activity of BMP or TGF-β (7, 10, 11).

Platelet-derived growth factors (PDGFs) are powerful mitogens and chemoattractants for cells of mesenchymal origins such as BMSC. The PDGF family is composed of four ligands (PDGF A, B, C, and D) and two transmembrane receptor tyrosine kinase subunits (PDGF receptor alpha [α-PDGFR] and PDGF receptor beta [β-PDGFR]) (12). PDGFs induce BMSC migration to the site of bone regeneration and support the expansion of osteoprogenitor cells as well as the promotion of angiogenesis, thereby indirectly facilitating bone formation (13, 14, 15). Consistently, preclinical and clinical studies have indicated that PDGF B supplementation *via* local application together with BMP-2 or TGF-β showed promising improvement in bone regeneration (7, 8, 16, 17, 18). However, its efficacy depends on the optimal concentrations of PDGF B relative to BMP-2 or TGF-β and the sequential schedule of PDGF delivery to BMP-2 rather than simultaneous administration, making it difficult to use as a clinical therapeutic intervention.

At present, little is known about the role of PDGF in the regulation of MSC differentiation into specific lineages. Previously, we showed that increased PDGF D expression is associated with both high Gleason score and tumor stage in prostate cancer. Prostate cancer cell–derived PDGF D promotes intraosseous tumor growth, associated with increased osteoblastic bone responses in mice (19, 20, 21). In this study, we investigated the molecular actions by which PDGF D regulates human bone marrow stem cell (hBMSC) migration, proliferation, and differentiation into osteoblasts or adipocytes.

## Results

### PDGF D induces BMSC proliferation, migration, and invasion

**Figure 1:**
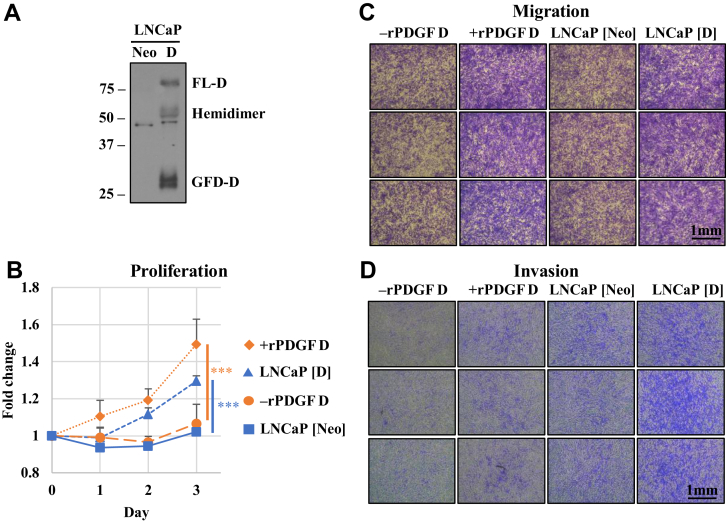
**PDGF D induces hBMSC proliferation, migration, and invasion.***A*, immunoblot analysis of PDGF D using conditioned media (CM) collected from control (Neo) and PDGF D-transfected LNCaP containing full-length dimer (FLD), hemidimer, and growth factor domain dimer (GFD) (61). *B*–*D*, WST-1 proliferation (*B*), transwell migration (*C*), and invasion (*D*) assays of hBMSC treated with CM from LNCaP (Neo) and LNCaP (D) or with serum-free media (SFM) without or with 1 nM rPDGF D. Migrated or invaded cells stain *blue*. hBMSC, human bone marrow mesenchymal stem cell; PDGF, platelet-derived growth factor; rPDGF, recombinant PDGF.

PDGF ligands are well known as powerful mitogens and chemoattractants for mesenchymal cells (12). We sought to confirm these properties of PDGF D using hBMSCs. As shown in Figure 1, recombinant PDGF (rPDGF) D as well as conditioned media (CM) collected from PDGF D-overexpressing LNCaP cells (22) significantly enhanced hBMSC proliferation, migration, and invasion compared with the controls (Fig. 1, *A*–*D*).

### PDGF D induces osteoblastic differentiation of BMSC

**Figure 2:**
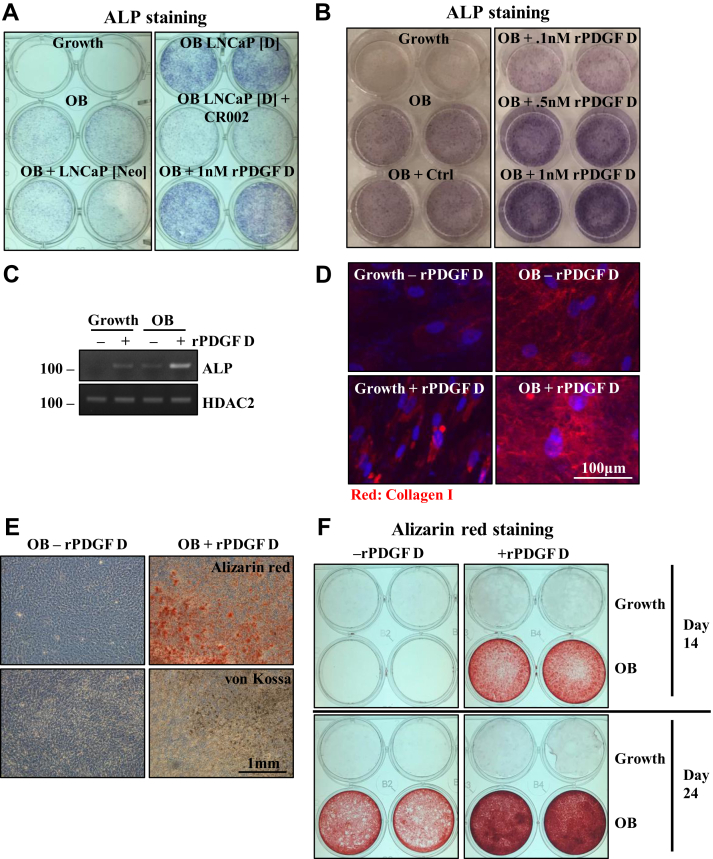
**PDGF D induces hBMSC osteoblastic differentiation.***A* and *B*, alkaline phosphatase (ALP) staining of hBMSCs cultured for 7 days (*A*) or 10 days (*B*) in growth or OB media containing CM from LNCaP (Neo) or LNCaP (D) without or with neutralizing AB CR002 or with rPDGF D proteins at indicated concentrations. *C*, RT–PCR analysis of ALP and HDAC2 (control). *D*, immunofluorescence staining of collagen I in hBMSCs cultured in growth or OB media with or without 1 nM rPDGF D for 3 days. *E* and *F*, Alizarin Red or von Kossa staining of hBMSCs cultured in the indicated media with or without 1 nM rPDGF D for 21 days (*E*). ALP, alkaline phosphatase; CM, conditioned media; hBMSC, human bone marrow mesenchymal stem cell; HDAC2, histone deacetylase 2; PDGF, platelet-derived growth factor; rPDGF, recombinant PDGF.

We previously demonstrated that intratibial injection of prostate carcinoma cells engineered to overexpress PDGF D induces overall osteoblastic bone reactions in mice (20, 21). Here, we examined the direct role of PDGF D on osteogenic differentiation of BMSC. Addition of CM collected from PDGF D-overexpressing LNCaP cells enhanced osteoblastic differentiation as evidenced by alkaline phosphatase (ALP) staining, a widely used marker for early osteoblast differentiation (Fig. 2*A*). The induction of ALP by PDGF D-overexpressing LNCaP CM was abolished by the neutralizing antibody CR002 against PDGF D (23) (a kind gift from Celldex Therapeutics), indicating the direct effect of PDGF D on osteoblastogenesis. We further examined the direct role of PDGF D on osteoblastogenesis using rPDGF D proteins. As shown in Figure 2*B*, PDGF D promoted osteoblastic differentiation in a dose-dependent manner. PDGF D induction of ALP expression was confirmed by RT–PCR analysis (Fig. 2*C*). Osteoblastic differentiation progresses through secretion of matrix proteins, such as collagen fiber, matrix maturation, and mineralization. Once mineralization is completed, calcium deposits can be detected. The ability of PDGF D to promote osteoblast maturation was confirmed by immunofluorescence (IF) staining of collagen I (Fig. 2*D*) as well as von Kossa and Alizarin staining for calcium deposits (Fig. 2, *E* and *F*).

### PDGF D inhibits BMSC differentiation into adipocytes

**Figure 3:**
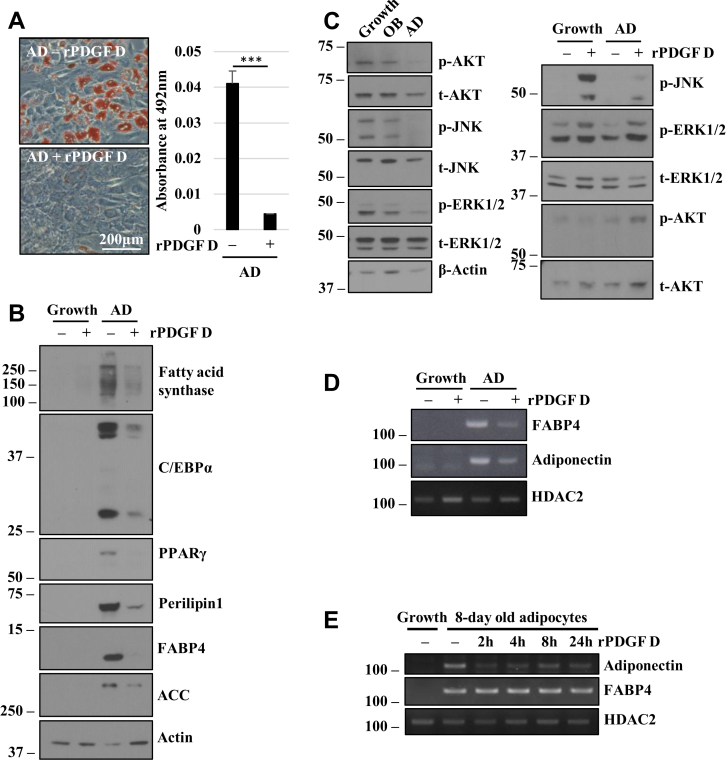
**PDGF D inhibits hBMSC adipocyte differentiation.***A*, Oil Red O staining (*left panel*) and quantitation (*right panel*) of hBMSCs cultured in adipocyte differentiation (AD) media, with or without 1 nM rPDGF D for 14 days. *B*, immunoblot analysis of indicated proteins in hBMSCs cultured in growth or AD media with or without 1 nM rPDGF D for 14 days. *C*, immunoblot analysis of indicated proteins in hBMSCs cultured in growth, OB, or AD media for 3 days (*left panel*) or AD media with and without 1 nM rPDGF D for 14 days (*right panel*). *D*, RT–PCR analysis of indicated mRNAs in hBMSCs cultured in growth or AD media with or without 1 nM rPDGF D for 14 days. *E*, RT–PCR analysis of indicated mRNAs in adipocytes cultured in AD media for 8 days and then treated with 1 nM rPDGF D for the indicated time points. hBMSC, human bone marrow mesenchymal stem cell; PDGF, platelet-derived growth factor; rPDGF, recombinant PDGF.

In addition to the osteoblastic lineage, BMSCs are known to differentiate into adipocytes. As shown in Figure 3*A*, rPDGF D significantly inhibited adipogenic differentiation of hBMSC as evidenced by Oil Red O staining of lipid droplets. Moreover, PDGF D downregulated CCAAT/enhancer binding protein alpha (C/EBPα) and peroxisome proliferator–activated receptor gamma, which are transcription factors critical for adipogenesis (24), as well as other adipogenic markers, such as the lipid droplet–associated protein perilipin 1, fatty acid–binding protein 4 (FABP4), and acetyl CoA carboxylase, a critical enzyme in the synthesis of fatty acids (25) (Fig. 3*B*). In addition, signaling molecules such as AKT, c-Jun N-terminal kinase (JNK), and extracellular signal–regulated kinase (ERK), which are often associated with cell proliferation, were downregulated during adipocyte differentiation (Fig. 3*C*). Interestingly, rPDGF D downregulation of adipocyte differentiation was associated with upregulation of these signaling molecules, especially AKT (Fig. 3*C*).

Next, we asked whether PDGF D can inhibit mRNA expression of regulators of adipogenesis even after hBMSC makes a commitment to the adipogenic lineage. First, we showed that constitutive stimulation of hBMSC with PDGF D in adipocyte differentiation culture media downregulated the mRNA expression of FABP4 and adiponectin, an autocrine factor that promotes differentiation from preadipocytes into adipocyte (26) (Fig. 3*D*). Interestingly, when adipogenic cells were treated with rPDGF D after culturing for 8 days in adipocyte differentiation media, downregulation of adiponectin mRNA was observed within 2 h while it had little effect on FABP4 mRNA levels (Fig. 3*E*). These results indicate that PDGF D can downregulate expression of some, if not all, adipogenic regulators even after BMSC lineage commitment to adipocytes.

### PDGF D-mediated differential regulation of BMSC differentiation into osteoblast *versus* adipocytes involves modulation of β-actin expression and polymerization

**Figure 4:**
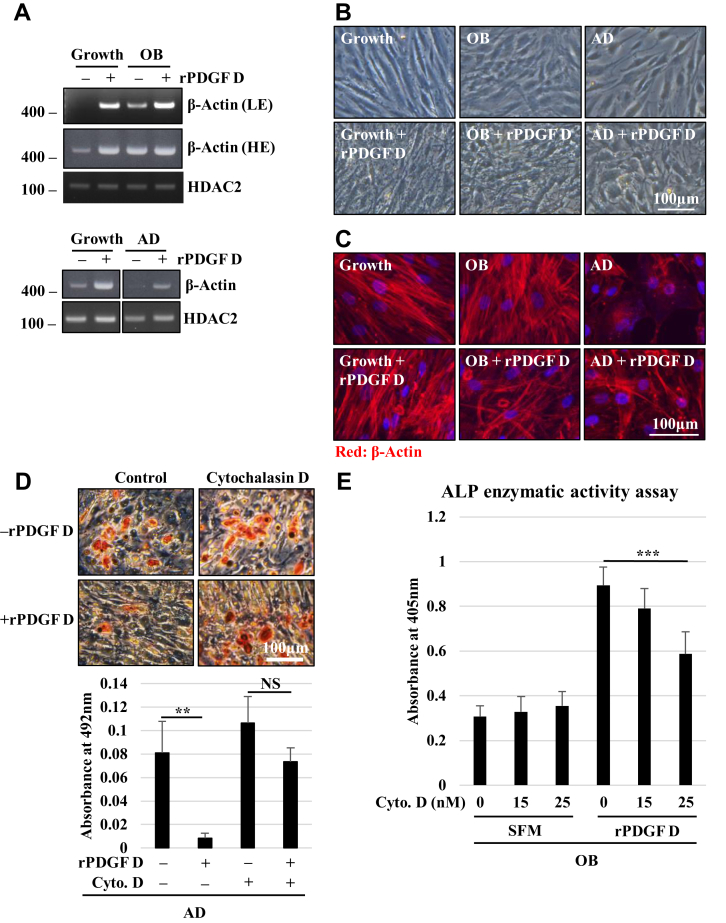
**PDGF D-regulated actin cytoskeleton is critical for the regulation of hBMSC differentiation into osteoblasts *versus* adipocytes.***A*, RT–PCR analysis of indicated mRNAs in hBMSCs cultured in growth and OB (*top panel*) or AD (*bottom panel*) with or without 1 nM rPDGF D for 3 days. *B* and *C*, bright-field microscopy (*B*) and immunofluorescence of β-actin (*C*) images of hBMSCs cultured in growth, OB, or AD media with or without 1 nM rPDGF D for 3 days. *D* and *E*, Oil Red O staining (*D*, *top panel*) and quantitation (*bottom panel*) and ALP enzymatic activity assay (*E*) of hBMSCs cultured in AD (*D*) or OB (*E*) media with or without 1 nM rPDGF D and with or without 20 nM cytochalasin D. Three replicates (*D*) and six replicates (*E*) were used for quantitation. AD, adipocyte differentiation; ALP, alkaline phosphatase; hBMSC, human bone marrow mesenchymal stem cell; HE, high exposure; LE, low exposure; PDGF, platelet-derived growth factor; rPDGF, recombinant PDGF.

Cytoskeletal changes serve as an intracellular signal that controls differentiation programs, and the actin cytoskeleton remodeling is critical in the regulation of cellular mechanics during osteogenic differentiation of stem cells (27). Interestingly, β-actin mRNA expression was induced in the osteogenic condition compared with the growth condition and with treatment of rPDGF D in both culture conditions (Fig. 4*A*, *upper panel*). Contrary to osteoblast differentiation, β-actin expression decreased during adipocyte differentiation (Fig. 4*A*, *lower panel*). BMSCs under growth condition exhibited a fibroblastic spindle-shaped morphology and grew in tight parallel formation (Fig. 4*B*), in agreement with previous reports (28, 29). This morphology was consistent with actin stress fibers running parallel along the axis of the cell (Fig. 4*C*). During osteoblast differentiation, the cells became rounder and flattened (Fig. 4*B*), and their actin network exhibited more robust crisscrossed stress fibers (Fig. 4*C*), consistent with previous reports (30, 31, 32). During adipocyte differentiation, BMSCs became more isolated because of lower proliferation, and the actin network becomes disrupted and downregulated (Fig. 4, *B* and *C*). These changes are thought to create space for lipid droplet storage (31). Interestingly, treatment with rPDGF D resulted in higher cell density as well as more crisscrossed and robust actin fibers in all three conditions (Fig. 4, *B* and *C*). Here, we asked whether PDGF D promotion of β-actin polymerization contributes to osteoblastic differentiation of BMSC and/or inhibition of adipogenic differentiation. When β-actin polymerization was inhibited by cytochalasin D, PDGF D was unable to either promote osteoblast differentiation or downregulate adipocyte differentiation (Fig. 4, *D* and *E*), indicating the significance of the PDGF D–β-actin axis in the regulation of BMSC differentiation.

### PDGF D treatment of hBMSC results in dramatic induction of β-PDGFR ubiquitination

**Figure 5:**
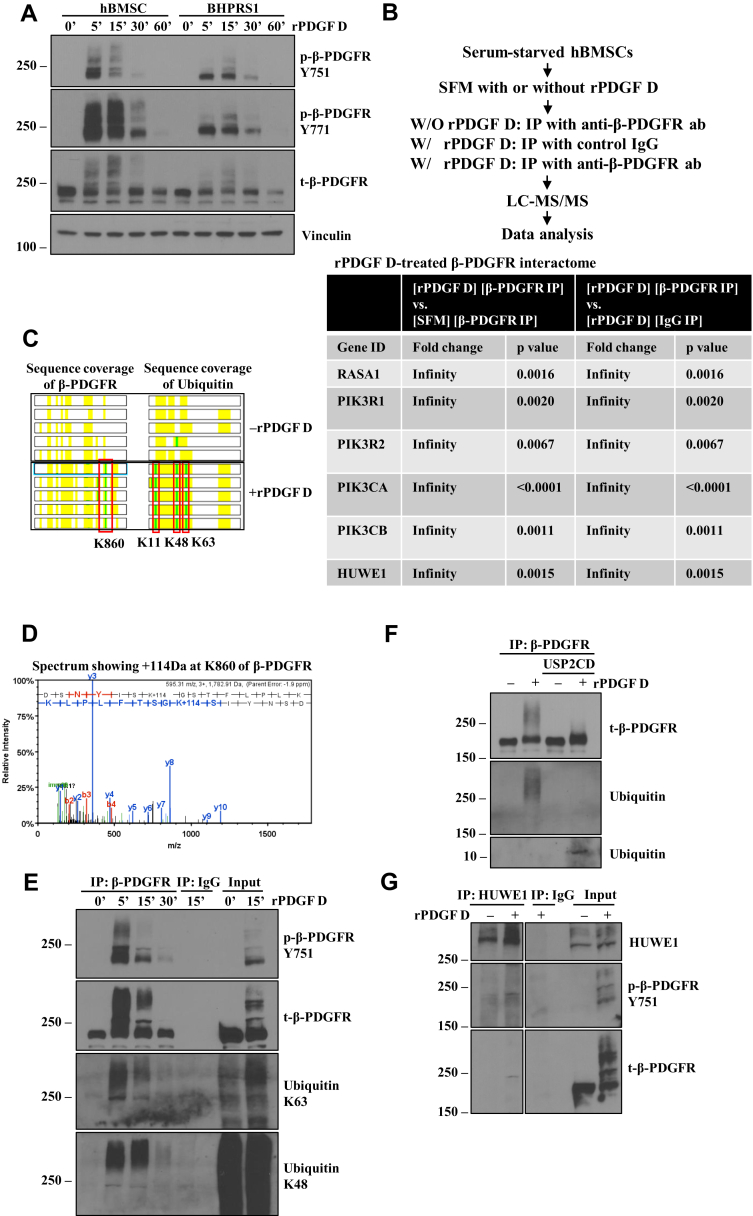
**Identification of the E3 ligase HUWE1 as an interactor of PDGF D-activated β-PDGFR in hBMSCs.***A*, immunoblot analysis of indicated proteins in hBMSC and BHPRS1 cells at indicated time points post treatments with 1 nM rPDGF D. *B*, immunoprecipitation as indicated, followed by LC–MS/MS analysis. Proteins that are significantly enriched in the immunoprecipitates of PDGF D-activated β-PDGFR as obtained from SFINX analysis are listed. *C*, the identified peptide sequences in the β-PDGFR and ubiquitin proteins in immunoprecipitates of β-PDGFR without (SFM) or with rPDGF D treatment are highlighted in *yellow*. The lysine residues with the Gly-Gly modification are marked in *green*. *D*, spectrum showing +114 Da at K860 of PDGF D-activated β-PDGFR. *E*, immunoblot analysis of immunoprecipitates from serum-starved hBMSCs treated with 1 nM rPDGF D using anti-β-PDGFR Ab or control IgG at indicated time points. *F*, USP2 catalytic domain (USP2CD)-mediated *in vitro* deubiquitination assay of immunoprecipitates of serum-starved hBMSCs treated without or with 1 nM rPDGF D for 10 min. *G*, immunoblot analysis of indicated proteins in immunoprecipitates from serum-starved hBMSCs treated without or with 1 nM rPDGF D using anti-HUWE1 Ab or control IgG. Total lysates (input) without or with rPDGF D treatment were also subjected to immunoblot analysis as a control. Ab, antibody; hBMSC, human bone marrow mesenchymal stem cell; HUWEI, HECT, UBA, and WWE domain–containing protein 1; IgG, immunoglobulin G; PDGF, platelet-derived growth factor; PDGFR, PDGF receptor; rPDGF, recombinant PDGF; USP2, ubiquitin-specific peptidase 2.

To characterize PDGF D-activated signaling complex critical for the regulation of hBMSC differentiation, we first examined the activation of its cell surface binding partner β-PDGFR. hBMSCs express an abundance of β-PDGFR, which is readily detectable by immunoblotting (Fig. 5*A*, total β-PDGFR). rPDGF D-activated β-PDGFR underwent massive upward molecular weight shifts associated with phosphorylation of the receptor in hBMSCs (Fig. 5*A*). This shift is much less prominent in the human prostate fibroblast cell line BHPRS1 (Fig. 5*A*) and the murine embryonic fibroblast cell line NIH3T3 (data not shown), suggesting that it is a unique response of β-PDGFR to its ligand PDGF D in hBMSCs. To characterize the molecular nature of the β-PDGFR signaling complex activated by rPDGF D in hBMSCs, the PDGF D-activated β-PDGFR complex was isolated by immunoprecipitation (IP) using anti–β-PDGFR antibody and also immunoglobulin G as a negative control for IP. As an additional control, the inactive β-PDGFR complex was isolated in the absence of rPDGF D treatment. These IP products were subjected to LC–MS/MS proteomic analysis. The protein complex of activated β-PDGFR was significantly enriched for Ras-GAP1 (GTPase-activating protein 1) and four PI3K isoforms, all known interactors of β-PDGFR (33) in comparison to inactivated β-PDGFR or control immunoglobulin G-interacting protein complex (Fig. 5*B*). Consistently, we detected PDGF D-induced phosphorylation of tyrosine residues 751 and 771 of β-PDGFR, known to create binding sites for PI3K and RasGAP, respectively (34, 35). Interestingly, active β-PDGFR-associated protein complex was also significantly enriched for HECT, UBA, and WWE domain–containing protein 1 (HUWE1), an E3 ubiquitin ligase. This suggests that the molecular weight shift may result from polyubiquitination of β-PDGFR. Further proteomic analysis revealed that the amino acid residue lysine-860 of β-PDGFR underwent a 114 Da-shift Gly-Gly modification indicative of an ubiquitination site (Fig. 5, *C* and *D*). This Gly-Gly modification is also detected in amino acid residues lysine 11, lysine 48, and lysine 63 of ubiquitin associated with activated β-PDGFR, suggesting that polyubiquitination of β-PDGFR utilizes lysine 11, lysine 48, and lysine 63 linkages (Fig. 5*C*). To confirm PDGF D-induced polyubiquitination of β-PDGFR, immunoprecipitates of β-PDGFR were subjected to immunoblot analysis using linkage-specific antiubiquitin antibodies. As shown in Figure 5*E*, while neither lysine 48 nor lysine 63 ubiquitin chain was detected in inactive β-PDGFR (time 0), both were readily detected in PDGF D-activated β-PDGFR. Next, we asked whether the molecular weight shift of β-PDGFR from ∼180 kDa to well above 250 kDa is mostly because of polyubiquitination. To this end, we performed an *in vitro* deubiquitinase assay in immunoprecipitates of β-PDGFR using the recombinant catalytic domain proteins of ubiquitin-specific peptidase 2 (USP2). High molecular weight species of β-PDGFR disappeared almost completely upon deubiquitination (Fig. 5*F*, *top* and *middle panels*) accompanied with accumulation of monoubiquitins each with a predicted molecular weight of 8.6 kDa (Fig. 5*F*, *bottom panel*). These results showed that β-PDGFR is heavily polyubiquitinated upon PDGF D treatment, possibly involving the E3 ligase HUWE1, which was identified as an interactor of PDGF D-activated β-PDGFR by proteomic analysis. We further confirmed their interactions by immunoblot analysis of β-PDGFR using immunoprecipitates of HUWE1 (Fig. 5*G*).

### HUWE1 prolongs cell surface residency of PDGF D-activated β-PDGFR in hBMSCs

**Figure 6:**
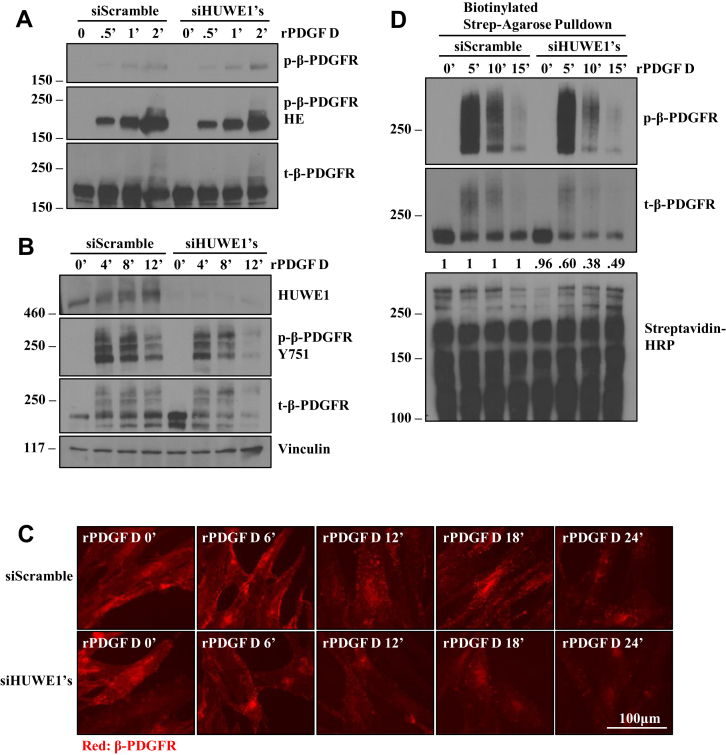
**HUWE1 mediates β-PDGFR ubiquitination and delays its internalization and degradation.***A* and *B*, immunoblot analysis of indicated proteins in hBMSCs with or without HUWE1 downregulation at indicated time points post treatments with 1 nM rPDGF D. *C*, immunofluorescence staining of β-PDGFR in hBMSCs in the absence or the presence of HUWE1 downregulation treated with 1 nM rPDGF D for up to 24 min. *D*, immunoblot analysis of indicated proteins using cell surface biotinylated hBMSC lysates, isolated using streptavidin–agarose bead, in the absence or the presence of HUWE1 downregulation, treated with 1 nM rPDGF D for the indicated period before cell surface biotinylation (*top panel*). Total biotinylated proteins are detected using streptavidin–HRP as a control (*bottom panel*). hBMSC, human bone marrow mesenchymal stem cell; HRP, horseradish peroxidase; HUWEI, HECT, UBA, and WWE domain–containing protein 1; PDGFR, platelet-derived growth factor receptor; rPDGF, recombinant PDGF.

**Figure 7:**
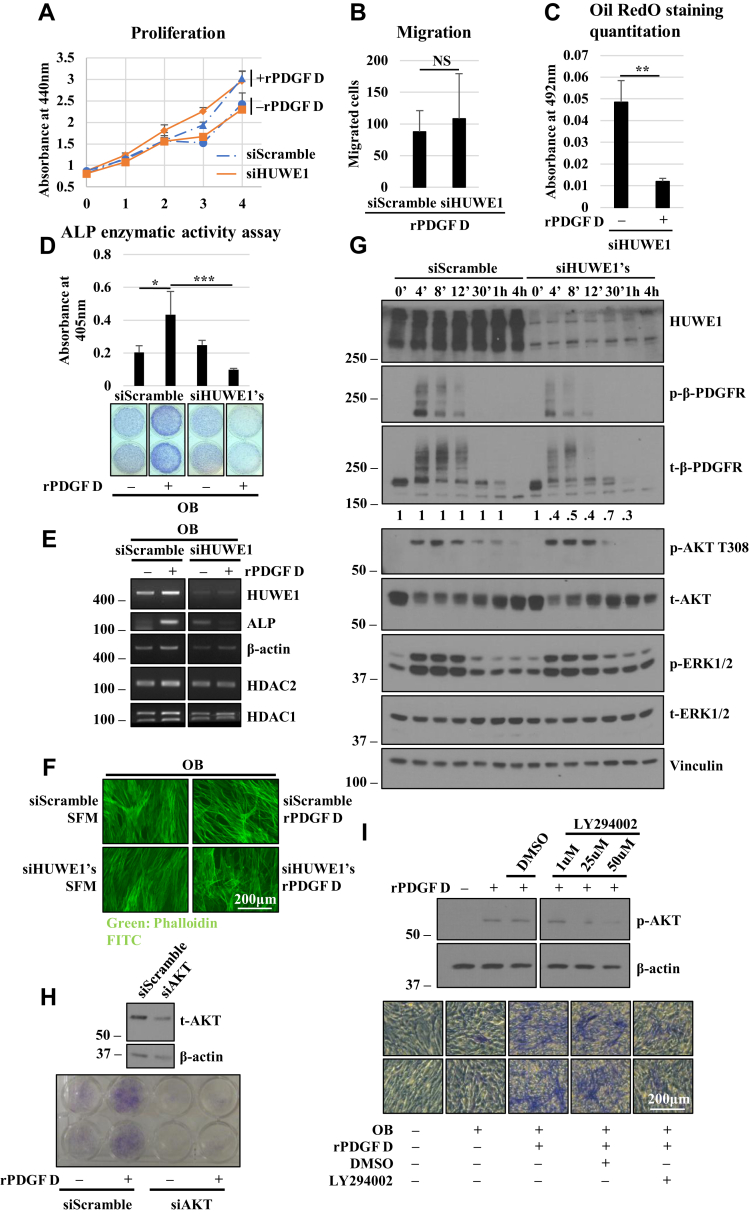
**The role of HUWE in PDGF D-regulated hBMSC proliferation, migration, and differentiation.***A* and *B*, WST-1 proliferation (*A*) and transwell migration (*B*) assays of hBMSCs without or with HUWE1 downregulation, treated with or without 1 nM rPDGF D. *C*, quantitation of Oil Red O staining in hBMSCs with or without HUWE1 downregulation, treated with or without 1 nM rPDGF D for 16 days. *D*, ALP enzymatic activity assay of hBMSCs with or without HUWE1 downregulation, treated with or without 1 nM rPDGF D. Five replicates were used for quantitation. *E* and *F*, RT–PCR analysis (*E*) or phalloidin staining (*F*) of hBMSCs with or without HUWE1 downregulation, treated with or without 1 nM rPDGF D for 3 days in OB media. *G*, immunoblot analysis of indicated proteins in hBMSCs with or without HUWE1 downregulation, treated with 1 nM rPDGF D for up to 4 h. *H* and *I*, immunoblot analysis of total or phospho Akt and β-actin in hBMSCs without or with Akt downregulation or LY294003 treatment (*top panels*). Control and Akt-downregulated hBMSC treated without or with rPDGF D (*H*) or hBMSCs in the presence of 25 μM LY294003 (*I*) are subjected to immunoblot analysis for total or phospho Akt and β-actin (*top panels*) or ALP staining following OB differentiation for 6 days (*bottom panels*). ALP, alkaline phosphatase; hBMSC, human bone marrow mesenchymal stem cell; HUWEI, HECT, UBA, and WWE domain–containing protein 1; PDGF, platelet-derived growth factor; rPDGF, recombinant PDGF.

To assess the functional significance of HUWE1 in activation and ubiquitination of β-PDGFR upon PDGF D treatments, we downregulated HUWE1 expression using siRNAs. Activation of β-PDGFR was detected as early as 30 s poststimulation of hBMSCs with rPDGF D regardless of HUWE1 expression (Fig. 6*A*), followed by ubiquitination of β-PDGFR detected at 4 min poststimulation (Fig. 6*B*). The degree of ubiquitination of β-PDGFR was reduced by approximately 50% by HUWE1 knockdown (KD) (Fig. 7*G*). Interestingly, the levels of active β-PDGFR were noticeably lower at 8 and 12 min post PDGF D treatments in HUWE1 KD cells compared with the control (Fig. 6*B*), an unexpected observation considering the well-known consequence of ubiquitination for the degradation of a target protein by the ubiquitin proteasome system (36). Upon ligand binding, PDGFRs are known to undergo receptor dimerization and cluster in patches on the cell membrane followed by receptor endocytosis and degradation (37). When we monitored the fate of PDGF D-activated β-PDGFR with or without HUWE1 KD by IF microscopy, HUWE1 KD led to the appearance of fewer and smaller β-PDGFR puncta and faster overall disappearance (Fig. 6*C*), consistent with results seen by immunoblot analyses (Fig. 6*B*). These results suggest that HUWE1-mediated polyubiquitination may slow down internalization and degradation of PDGF D-activated β-PDGFR, thereby extending the duration of β-PDGFR signaling on the cell surface. To determine whether HUWE1 affects internalization of β-PDGFR, we activated β-PDGFR with rPDGF D and labeled and purified cell surface proteins using Sulfo-NHS-Biotin (Thermo Fisher Scientific; catalog no.: 21217) and streptavidin–agarose beads (Sigma; catalog no.: S1638). The level of cell surface β-PDGFR was greatly reduced at 10 and 15 min postactivation in HUWE1 KD hBMSC cells compared with the control cells (Fig. 6*D*). Taken together, these results showed that HUWE1 recruitment to the PDGF D-activated β-PDGFR complex contributes to the polyubiquitination of β-PDGFR and delays its internalization and degradation.

### HUWE1 is critical for PDGF D-induced osteoblastic differentiation of hBMSC involving Akt

Increasing evidence suggested that relatively subtle differences in the duration and strength of the RTK signal can lead to different cellular processes (*e.g.*, cell proliferation *versus* differentiation) (38, 39, 40). Thus, we asked whether HUWE1-regulated duration of PDGF D-activated β-PDGFR signal on the cell surface plays a role in the regulation of hBMSC proliferation, migration, and/or differentiation into a specific lineage. HUWE1 KD had little effect on hBMSC migration or proliferation (Fig. 7, *A* and *B*). In the presence of HUWE1 downregulation, PDGF D activation of β-PDGFR was still able to inhibit adipogenic differentiation of hBMSCs (Fig. 7*C*). However, PDGF D activation of β-PDGFR failed to promote osteoblastic differentiation in HUWE1 KD hBMSCs as assessed by ALP staining and ALP enzymatic activity (Fig. 7*D*). PDGF D-induced ALP mRNA expression was dependent on HUWE1, whereas the upregulation of β-actin was independent of HUWE1 (Fig. 7*E*). The ability of the PDGF D/β-PDGFR signaling axis to promote robust remodeling of the actin cytoskeleton was also independent of HUWE1 (Fig. 7*F*).

In search of HUWE1-dependent signaling pathways critical for PDGF D promotion of osteoblastogenesis, we observed intense but shorter-lived Akt activation by PDGF D in HUWE1 KD cells compared with the control cells, whereas the kinetics of ERK activation were barely affected by HUWE1 KD (Fig. 7*G*). The functional significance of Akt in PDGF D-promoted osteoblastic differentiation of hBMSCs was assessed by siRNA-mediated KD approach as well as using LY294002, a pharmacological inhibitor of its upstream activator PI3K. Both approaches effectively abolished PDGF D-induced osteoblastogenesis (Fig. 7,*H* and *I*), indicating the significance of the β-PDGFR/HUWE1/PI3K/Akt signaling pathway for PDGF D-mediated osteoblastic differentiation of hBMSCs.

## Discussion

The classic members of the PDGF family PDGF A and B were identified almost half a century ago as potent mitogens and chemoattractants for cells of mesenchymal origin (41). Mice deficient of PDGF A or B show severe defects in organ development and die either as embryos or shortly after birth, demonstrating their indispensable functions during mouse development (42, 43). In the early 2000s, two new members of the PDGF family, PDGF C and D, were discovered (44, 45). While PDGF A and B are secreted as active growth factor homodimer or heterodimer, PDGF C and D are secreted as latent growth factor homodimers containing the N-terminal CUB and C-terminal growth factor domains. Extracellular serine protease–mediated removal of the CUB domain is required for the growth factor domain dimers of PDGF C and D to activate their cognate receptor α and β, respectively (45, 46). Using the growth factor domain dimers of rPDGF D, the present study demonstrates a novel function of PDGF D in bone formation by simultaneously promoting the commitment of osteoblastic differentiation of BMSCs while inhibiting their commitment to adipogenesis. Unlike mice deficient of PDGF A or B, mice-deficient PDGF D exhibit only a mild vascular phenotype (47). Thus, PDGF D is unlikely to play a critical role in bone formation during development but may contribute to bone healing in adults or to disease-associated bone reactions. Indeed, our previous study demonstrated that prostate carcinoma–produced PDGF D induces osteoblastic bone reactions and promote intraosseous tumor growth in mice and that treatments with cediranib (an inhibitor of PDGFR/vascular endothelial growth factor receptor) significantly reduced the trabecular bone levels (∼75% reduction) ((20, 21) and Fig. S1). In addition to its known functions in recruiting BMSCs to the site of bone regeneration, expanding BMSC populations, and promoting angiogenesis, the ability of PDGF D to induce osteoblastic differentiation of BMSC may make it an ideal therapeutic agent for bone regeneration/healing. It would be of significance to evaluate the potential use of rPDGF D to promote bone repair/regeneration by itself and/or in conjunction with established bone-healing agents such as BMP-2 or TGF-β.

To our knowledge, this is the first report that PDGF can directly regulate the commitment of MSC differentiation into a specific lineage. The functions of the classic PDGF family members PDGF A and B during embryogenesis and development are mediated mostly by their roles in cell–cell communication. Epithelium-derived PDGF A and B activates their cognate receptors α-PDGFRs and β-PDGFRs in surrounding stromal cells, thereby supporting organ development processes (48). Similarly, activation of PDGFRs in vascular smooth muscle cells or pericytes is critical for vasculogenesis and angiogenesis during development (49). PDGF-mediated inhibition of adipogenic differentiation was previously suggested by inducing MSC proliferation, thereby indirectly inhibiting MSC differentiation into adipocytes (50). Importantly, the present study provides evidence that PDGF D-activated β-PDGFR prevents MSCs from their adipogenic differentiation by maintaining the actin cytoskeleton as well as downregulation of adipogenic gene expression. In addition to inhibition of adipogenesis, PDGF D promotes osteogenic differentiation of MSCs by inducing remodeling of the actin cytoskeleton and upregulation of osteogenic gene expression.

Changes in cellular morphology and the actin cytoskeleton of hBMSCs during osteogenic differentiation have been reported previously (30, 51). In undifferentiated hBMSCs, actin fibers run in organized parallel formation along the cell axis to maintain a fibroblastic spindle-shaped morphology (51). During differentiation into osteoblasts, the actin cytoskeleton adopts a more disorganized crisscrossing pattern that is consistent with a cuboidal and spreading cellular morphology optimal for matrix deposition (30, 31, 51). During hBMSC differentiation, cell shape changes regulate RhoA (Ras homolog family member A, a protein in the Rho family of GTPases) activity, which regulates ROCK (Rho-associated protein kinase) activity, which in turn regulates myosin-generated cytoskeleton tension through phosphorylation of myosin light chain (31). This signaling axis has been shown to directly regulate MSC differentiation into osteoblasts. Interestingly, PDGF signaling cascade induces RhoA and ROCK activities and regulates actin polymerization (52, 53, 54). These studies and our data reported in this article suggest that PDGF D induces osteogenic differentiation of hBMSCs through the upregulation of cytoskeleton tension possibly involving RhoA–ROCK–MCL–actin networks.

The present study identified the E3 ligase HUWE1 as a critical regulator of PDGF D-induced β-PDGFR for the regulation of osteogenic differentiation of hBMSCs. Ligand-induced ubiquitination of β-PDGFR was previously shown by the Cbl family of E3 ubiquitin ligases, which act primarily as a negative regulator of β-PDGFR signaling by facilitating receptor internalization, proteasomal degradation, and termination of mitogenic and chemotactic signaling (55, 56). In those studies, ubiquitination of β-PDGFR was detected by immunoblot analysis of immunoprecipitates of the β-PDGFR or ectopically overexpressed β-PDGFR with a tag in the presence of proteasome inhibitors such as MG132. In our study, HUWE1-mediated polyubiquitination of β-PDGFR was readily detected by immunoblot analysis of whole cell lysates even in the absence of an inhibitor of the ubiquitin–proteasome system. In contrary to the Cbl family, HUWE1-mediated β-PDGFR prolongs the presence of β-PDGFR on the cell surface following its activation, thereby extending its osteogenic signals.

The large extent of the ubiquitination process on β-PDGFR in hBMSCs, exhibited by molecular weight shift from ∼180 kDa to >460 kDa, may occur in more than one site in β-PDGFR. Our proteomic analysis identified lysine 860 in β-PDGFR as an ubiquitination site. Since the sequence coverage of β-PDGFR in our analysis was about 30% (Fig. 5*C*), there could be other lysine residues in the intracellular domains of β-PDGFR that are ubiquitinated. A search of the web-based bioinformatics resource PhosphositePlus showed four additional lysine resides (K645, K707, K762, and K841) in β-PDGFR that carry the Gly-Gly modification identified through high-throughput screenings by other investigators (57). In our analysis reported here, lysine 707 did not undergo Gly-Gly modification, and K645, K762, and K841 residues were not covered by our proteomic screening. Although the present study clearly demonstrated the functional significance of HUWE1 in the regulation of β-PDGFR-mediated osteogenic differentiation of hBMSCs, HUWE1 may not be the sole E3 ligase responsible for PDGF D-induced polyubiquitination of β-PDGFR in hBMSCs, considering a significant level of ubiquitinated β-PDGFR remained in HUWE1 KD cells (Fig. 6*B*). Ubiquitin itself can be elongated through seven lysine residues and an N terminus, and the type of lysine linkage is essential in determining the fate of the substrate (58, 59). An analysis of our proteomic data revealed that polyubiquitination of β-PDGFR utilized at least three types of lysine linkages (K11, K48, and K63). It remains to be investigated whether the specific order and function of these lysine linkages are critical for HUWE1-regulated retention of β-PDGFR on the cell surface or whether ubiquitin modifications by the PDGF D-activated β-PDGFR signaling complex such as phosphorylation results in alterations in the tertiary structure, thereby stabilizing the complex and activating unique signal transduction pathways.

## Experimental procedures

### hBMSC culture and differentiation

hBMSCs were purchased from Lonza (catalog no.: PT-2501) and grown in MesenPro RS media (Thermo Fisher Scientific; catalog no.: 12746012). Accutase (Thermo Fisher Scientific; catalog no.: A110501) was used as a cell dissociation reagent, and hBMSCs were frozen in supplement-free MesenPro RS media containing 10% dimethyl sulfoxide and 4% human albumin for storage. To induce adipogenic differentiation, cells were grown in adipogenic differentiation medium (Thermo Fisher Scientific; catalog no.: A10410-01). To induce osteoblastic differentiation, cells were grown in complete MesenPro RS media supplemented with 10 mM β-glycerol phosphate (Sigma; catalog no.: 50020), 50 μg/ml ascorbic acid (Sigma; catalog no.: A4403), and 100 nM dexamethasone (Sigma; catalog no.: D4902) or in osteogenic media purchased from Lonza (catalog no.: PT-3924).

### Staining and quantification for adipogenic differentiation

To detect lipid droplets, cells were washed with PBS, fixed with 4% paraformaldehyde (PFA) for 10 min at room temperature, washed with water and then with 60% isopropanol and stained with Oil Red O (Sigma; catalog no.: O1391) for 10 min. Cells were washed five times with copious amounts of water and photographed at 5× magnification using a Leica DMi1 microscope. For quantification, cells were washed three times with 60% isopropanol, and then Oil Red O dye was eluted using 100% isopropanol. Absorbance of the eluate was measured at 492 nm.

### Staining for osteoblastic differentiation

ALP staining was performed according to the manufacturer’s instructions (Sigma; catalog no.: 85L2-1KT). For Alizarin red staining, cells were washed with PBS, fixed in 4% PFA, washed with double-distilled (DD) water, and incubated in Alizarin red solution (Sigma; catalog no.: TMS-008-C) for 5 min at room temperature. Cells were then washed three times with DD water, let dry, and photographed. For von Kossa staining, cells were washed with PBS, fixed in 4% PFA, then washed two times with water, and incubated in 1% silver nitrate (Aldon Corporation; catalog no.: AD-20315) solution under UV light for 1 h. Cells were washed two times with DD water, followed by a 5% sodium thiosulfate (Sigma; catalog no.: 217263) wash for 5 min and then photographed.

### Generation of CM

CM from LNCaP cells overexpressing PDGF D was generated as previously described (22).

### Cell proliferation, migration, and invasion assays

For cell proliferation, hBMSCs were plated in a 96-well plate and treated with serum-free media with or without 1 nM rPGDF D protein (R&D Systems; catalog no.: 1159-SB-025). Viable cells were assayed using WST-1 (Sigma; catalog no.: 11644807001) following the manufacturer’s instructions. Cell migration assay was performed as described previously (22). For cell invasion, the 24-well Transwell inserts (Fisher Scientific; catalog no.: 07-200-174) were coated with rat tail Collagen I (Fisher Scientific; catalog no.: CB-40236) at 10 μg/cm^2^.

### RT–PCR

RT–PCR analyses were performed as previously described (20). Primers were used as follows: β-actin (489 bp), forward primer—ACAGAGCCTCGCCTTTGC and reverse primer—GAGGCGTACAGGGATAGCAC; ALP (135 bp), forward primer—AGCTGAACAGGAACAACGTGA and reverse primer—CTTCATGGTGCCCGTGGTC; FABP4 (187 bp), forward primer—ACTGGGCCAGGAATTTGACG and reverse primer—AACTCTCGTGGAAGTGACGC; Adiponectin (185 bp), forward primer—CCATCTCCTCCTCACTTCCA and reverse primer—GAGTCGTGGTTTCCTGGTCAT; HUWE1 (437 bp), forward primer—TCCTTCGCTTTGCAGAGACT and reverse primer—GTTGCAGTGGGAAGATGGAT; histone deacetylase 1 (168 bp), forward primer—GGAAATCTATCGCCCTCACA and reverse primer—AACAGGCCATCGAATACTGG; histone deacetylase 2 (151 bp), forward primer—TCATTGGAAAATTGACAGCATAGT and reverse primer—CATGGTGATGGTGTTGAAGAAG; RUNX2 (143 bp), forward primer—GGTTAATCTCCGCAGGTCACT and reverse primer—CACTGTGCTGAAGAGGCTGTT; OSX (326 bp), forward primer—GTGTCTACACCTCTCTGGACAT and reverse primer—CTTGGGTTTATAGACATCTTGG; ColI (144 bp), forward primer—ACCGCCCTCCTGACGCAC and reverse primer—GCAGACGCAGATCCGGCAG.

### IF staining

IF staining was performed as previously described (20).

### IP

Serum-starved hBMSCs were treated with 1 nM rPDGF D for 10 min and lysed. Cell lysates were incubated with primary antibody at 4 °C overnight on a rotating shaker. Protein A agarose slurry (Thermo Fisher Scientific; catalog no.: 20365) was added for 2 h. Agarose beads were washed five times with cold radioimmunoprecipitation assay (RIPA) buffer and resuspended in denaturing sample buffer. Samples were heated at 95 ^°^C for 5 min, and the supernatants were extracted for further analysis.

### Proteomic analysis

IP products were precipitated in 5 volume 100% methanol—1 mM acetic acid at −20 °C overnight, followed by centrifugation at 17,000*g* at 4 °C for 20 min, rinsed with 100 ml methanol–acetic acid, and pelleted again. Supernatant was removed, and the pellets were dried using speed-vac and resolubilized in 0.375% deoxycholate, 50 mM triethylamine bicarbonate, and 1.25× PBS using the Q Sonica sonicator. As a control, a tube containing no protein was processed in parallel to gauge background contamination. Samples were then reduced with 5 mM DTT and alkylated with 15 mM iodoacetamide in the dark at room temperature. Excess iodoacetamide was quenched with an additional 5 mM DTT. An overnight digestion was performed with sequencing-grade trypsin (Promega) in 40 mM triethylamine bicarbonate, 0.3% deoxycholate, and 0.3 M urea in PBS. The next day, the samples were incubated in 1% formic acid on ice for 30 min, followed by centrifugation at 17,000*g* at 4 °C for 10 min. One-fourth of the supernatant was analyzed by mass spectrometry. The peptides were separated by reversed-phase chromatography (Acclaim PepMap100 C18 column; Thermo Fisher Scientific), followed by ionization with the Nanospray Flex Ion Source (Thermo Fisher Scientific), and introduced into a Q Exactive mass spectrometer (Thermo Fisher Scientific). Abundant species were fragmented with high-energy collision-induced dissociation. Data analysis was performed using Proteome Discoverer 2.1 (Thermo Fisher Scientific), which incorporated the Sequest algorithm (Thermo Fisher Scientific). The UniProt_Hum_Compl_20180406 database (20,260 entries) was searched for human protein sequences, and a reverse decoy protein database was run simultaneously for false discovery rate (FDR) determination. The Proteome Discoverer files were loaded into Scaffold (Proteome Software) for distribution. Sequest was searched with a fragment ion mass tolerance of 0.02 Da and a parent ion tolerance of 10 ppm (parts per million) with trypsin allowing up to two missed cleavage. Carbamidomethylation of cysteine was specified in Sequest as a fixed modification. Deamidation of asparagine and glutamine, oxidation of methionine, and acetylation of the N terminus were specified in Sequest as variable modifications. About 464 total proteins were identified from 82,584 MS2 spectra. Minimum protein identification probability was set at an FDR of 1% for both proteins and peptides, and two unique peptides were required for proteins. FDR was determined using the Scaffold Local FDR algorithm. Scaffold uses the Protein Prophet algorithm to establish protein identification probabilities (60). Protein abundance was based on total spectral counts assigned to the protein. Ubiquitination sites on peptides were determined by a mass shift of 114 Da for lysine residues that is indicative of the addition of the Gly-Gly residues from the carboxyl terminus of ubiquitin. Raw mass spectrometric data (exclusive peptide count) were analyzed using the SFINX program to identify protein–protein interactions. β-PDGFR treated with PDGF D was used as “bait.”

### Cell surface biotinylation

Serum-starved hBMSCs were treated with 1 nM rPDGF D, washed three times with ice-cold PBS, pH 8.0, and incubated with ice-cold freshly prepared 2 mM Sulfo-NHS-Biotin in PBS, pH 8.0, for 30 min at 4 ^°^C. Cells were then washed three times with 100 mM glycine in PBS, washed once in regular PBS, and then lysed with RIPA buffer. Equal amounts of proteins in each sample were incubated with streptavidin–agarose beads for 3 h at 4 ^°^C, centrifuged, washed three times with RIPA buffer, and boiled in reducing sample buffer for 5 min. The supernatants were removed for immunoblot analysis.

### *In vitro* deubiquitination assay

Immunoprecipitates were washed with PBS, centrifuged, and incubated with 5 nM recombinant catalytic domain of USP2 (USP2CD; R&D Systems; caalog no.: E-504-050) at 37 ^°^C for 2 h in reaction buffer (50 mM Tris–HCl, 50 mM NaCl, 1 mM EDTA, 10 mM DTT, 5% glycerol) at pH 8.0 with periodic shaking. Reducing sample buffer was added, followed by boiling at 95 ^°^C for 5 min, and samples were subjected to immunoblot analysis.

### Immunoblot analysis

SDS-PAGE and immunoblot analysis of samples were performed using the following antibodies: custom-made PDGF D (22), fatty acid synthase (Cell Signaling Technologies [CST]; catalog no.: 3180), C/EBPα (CST; catalog no.: 8178), peroxisome proliferator–activated receptor gamma (CST; catalog no.: 2435), perilipin (CST; catalog no.: 9349), FABP4 (CST; catalog no.: 2120), acetyl CoA carboxylase (CST; catalog no.: 3676), β-actin (CST; catalog no.: 4970), p-AKT (CST; catalog no.: 9275), AKT (CST; catalog no.: 9272), p-JNK (CST; catalog no.: 4668), JNK (CST; catalog no.: 9258), p-ERK1/2 (CST; catalog no.: 9101), ERK1/2 (CST; catalog no.: 9102), p-β-PDGFR Y751 (CST; catalog no.: 3161), p-β-PDGFR Y771 (CST; catalog no.: 3173), β-PDGFR (CST; catalog no.: 3169), vinculin (Sigma; catalog no.: V9131), ubiquitin (CST; catalog no.: 3936), ubiquitin-K48 (Abcam; catalog no.: ab140601), ubiquitin-K63 (Abcam; catalog no.: ab179434), HUWE1 (Abcam; catalog no.: ab70161), and streptavidin–horseradish peroxidase (Abcam; catalog no.: ab7403).

### Statistical analysis

Unpaired Student’s *t* test was used to assess the statistical significance of the difference between two groups. ∗*p* < 0.05. ∗∗*p* < 0.01. ∗∗∗*p* < 0.001. NS stands for “not significant.”

## Data availability

Data will be made available upon request.

## Supporting information

This article contains supporting information.

## Conflict of interest

H.-R. C. K. has patent pending to Wayne State University. The other authors declare that they have no conflicts of interest with the contents of this article.

## Supporting information

ProteomicSupplementalTable.xltm

SupplementalFigures 4.21.22.Pdf
