# Supplementary material for: E3 ligase HUWE1 promotes PDGF D-mediated osteoblastic differentiation of mesenchymal stem cells by effecting polyubiquitination of β-PDGFR
Source: J Biol Chem. 2022 Apr 25;298(6):101981. doi: 10.1016/j.jbc.2022.101981 (PMC9133640; doi:10.1016/j.jbc.2022.101981)
Supplement: SupplementalFigures 4.21.22.Pdf [file mmc2.pdf]

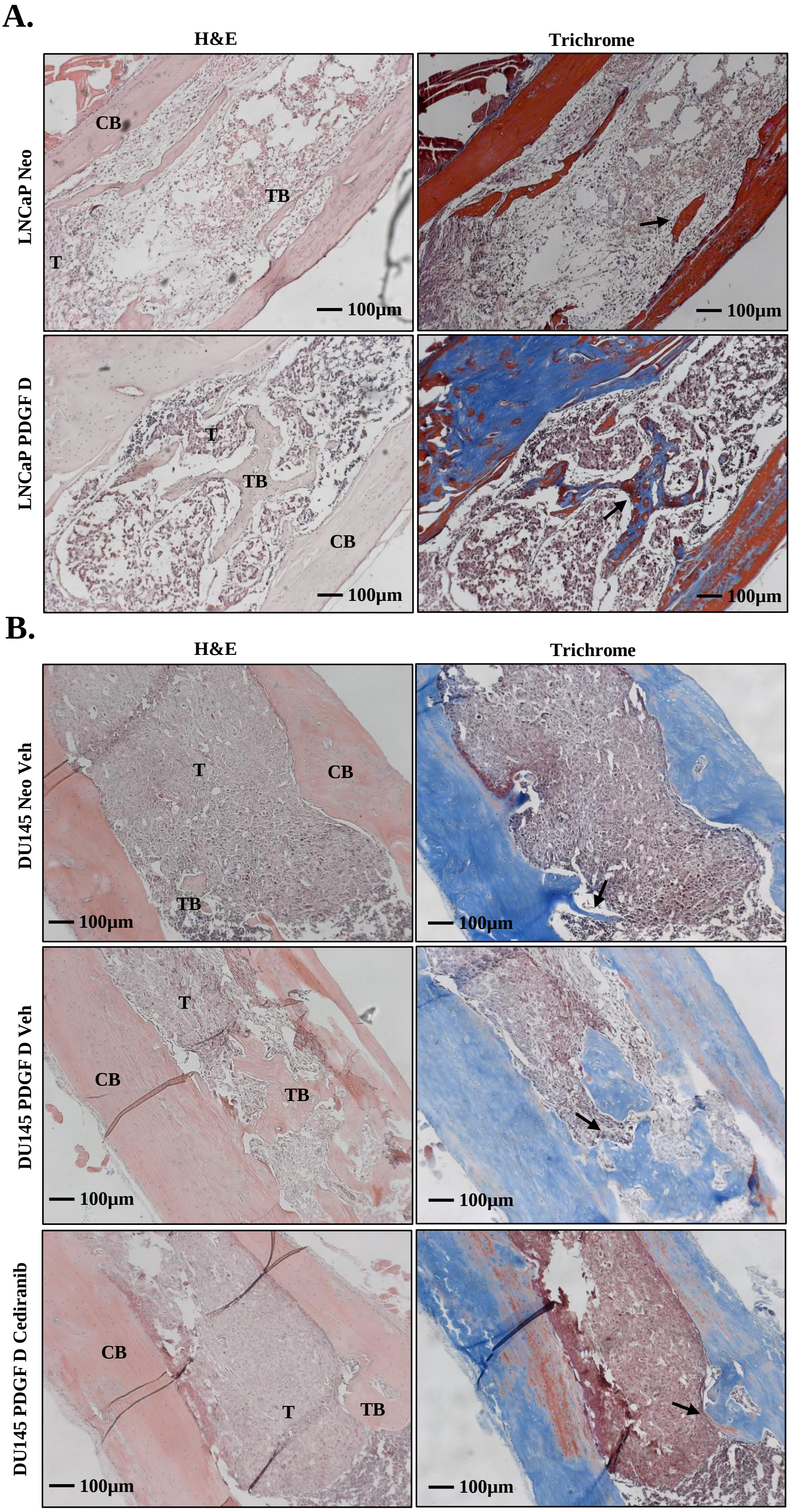

Supplementary Figure 1. PDGF D/β-PDGFR signaling mediates bone formation in vivo. (A) Control (Neo) and PDGF D overexpressing LNCaP cells were injected into the tibia of male SCID mice. At week 18 post injection, tibia were resected and decalcified, then paraffin-embedded for H&E and Trichrome staining. (B) Control (Neo) and PDGF D overexpressing DU145 cells were injected into the tibia of male SCID mice. Two weeks post injection, mice were randomized into vehicle or Cediranib (b-PDGFR/VEGFR inhibitor) treatment groups. At week 9 post injection, tibia were decalcified then paraffin-embedded for H&E and Trichrome staining. CB, cortical bone; T, tumor; TB, trabecular bone.
